# Investigation on Crystallization and Magnetic Properties of (Nd, Pr, Ce)_2_Fe_14_B/α-Fe Nanocomposite Magnets by Microwave Annealing Treatment

**DOI:** 10.3390/ma14112739

**Published:** 2021-05-22

**Authors:** Zhanyong Wang, Changping Shangguan, Zemin Wang, Tianpeng Wang, Lianbo Wang, Min Liu, Yanli Sui

**Affiliations:** 1 Department of Materials Science and Engineering, Shanghai Institute of Technology, Shanghai 201418, China; harrisguan@126.com (C.S.); wangtianpeng0528@163.com (T.W.); wanglianbo021@hotmail.com (L.W.); liumin110ok@163.com (M.L.); 2 Department Key Laboratory for Advanced Metals and Materials, University of Science and Technology Beijing, Beijing 100083, China; yls@ustb.edu.cn

**Keywords:** (Nd; Pr; Ce)_2_Fe_14_B/α-Fe, nanocomposite magnets, microwave-assisted annealing

## Abstract

In the present work, the structures and magnetic properties of (Nd, Pr, Ce) _2_Fe_14_B/α-Fe nanocomposite magnets were thoroughly investigated. The microwave annealing was applied to achieve a uniform heating effect and uniform grains. Microwave annealing is more favorable to obtain α-Fe phase than conventional annealing, which leads to the enhanced coercivity of hysteresis loops. The coercivity of nanocomposite magnets was 245 kA/m after annealing at 2000 W for 10 min.

## 1. Introduction

Nd_2_Fe_14_B/α-Fe nanocomposite magnets have attracted considerable research attention owing to the highest theoretical maximum energy product [1,2,3]. As previously reported, adjustment of the alloy composition and control of the crystallization process, especially the substitution of the Fe and Nd elements [4,5,6] was applied to improve the microstructure and properties [7,8]. Many elements (such as Pr, Y, etc.) have been applied to tailor the grain size and the crystallization phase [9,10,11]. As two effective rare earth elements, Pr and Ce have been widely used to substitute the Fe and Nd elements of Nd_2_Fe_14_B/α-Fe nanocomposite magnets and adjust their magnetic properties. Pr_2_Fe_14_B and Ce_2_Fe_14_B have the same crystal structure as the Nd_2_Fe_14_B, and the replacement of Ce element to Nd is beneficial to reduce the cost of RE-Fe-B magnets [12]. Nevertheless, the substitution of Ce for Nd deteriorates the magnetic properties because of the formation of Ce_2_Fe_14_B with low intrinsic magnetic properties [13], so in this research, the addition of Pr element is necessary, because the Pr substitution for the Nd improves the hard magnetic properties of the nanocomposite magnets [14]. The magnetic properties are significantly improved after the substitution of Ce and Pr elements to Nd [15]. Diffusion-processing is the typical method to fabricate high magnetic performance Nd-Pr-Ce-Fe-B sintered magnets [16]. For nanocomposite magnets with Ce and Pr substitution, the uniform and fine microstructure become the critical factor in improving the magnetic properties.

As is well known, the optimization of the annealing process is one of the effective ways to obtain uniform and fine microstructure of nanocomposite magnets [17,18]. The microwave heating process has shown significant advantages against conventional heating procedures in structure controlling. Microwave heating can heat materials to high temperatures with ultra-fast heating rates, short time, and high energy efficiency [19,20,21,22,23,24]. A higher heating rate favors obtaining uniform and refined grains after the crystallization process [25], enhancing coercivity, and also adjusts the proportion of hard and soft phases. Thus, the application of microwave annealing may show great benefit for the improvement of magnetic properties of Nd_2_Fe_14_B/α-Fe nanocomposite magnets.

The contributions of our research are highlighted as follows: we crystallized amorphous (Nd, Pr, Ce)_2_Fe_14_B/α-Fe nanocomposite ribbons by microwave heating treatment and investigated their magnetic properties, which has rarely been reported before. 

## 2. Materials and Methods

### 2.1. Materials

The ingots with nominal composition (Nd_0.__525_Pr_0.__175_Ce_0.__3_)_9_Fe_64.__5_Co_3_Cu_0.__5_Ti_1_B_22_ ribbons were prepared by arc-melting under a high-purity Ar atmosphere. The sizes of small pieces of ingots are 5–7 mm, they were re-melted and spun onto a copper roll, and the wheel speed 22 m/s.

### 2.2. Methods

To study the effect of microwave-assisted annealing on crystallization, we used a quartz tube with a vacuum of 5 × 10^−3^ Pa to seal the as-spun ribbons, and apply direct microwaves on ribbons at 650 °C for 10 min under the power of 800, 1500, and 2000 W, respectively. The HAMiLab-V3 microwave furnace (Beijing Yiye Lantian Technology Co., Ltd., Beijing, China) with a frequency of 2.45 GHz is experimental equipment. The (Nd_0.__525_Pr_0.__175_Ce_0.__3_)_9_Fe_64.__5_Co_3_Cu_0.__5_Ti_1_B_22_ as-spun ribbons were heated at a rate of 5, 20, and 40 °C/min. We used a differential scanning calorimeter (DSC, (Shanghai Precision Scientific Instrument Co., Ltd., Shanghai, China) to characterize the as-spun ribbons, and X-ray diffraction (XRD) with Cu-Kα radiation to determine the structure of the as-spun and annealed ribbons. A vibrating sample magnetometer (VSM, (YP Magnet Technology Development Co., Ltd.) is used to measure the magnetic properties of the ribbons with a maximum applied magnetic field of 1.8 T.

## 3. Results and Discussion

### 3.1. Crystallization Process

**Figure 1:**
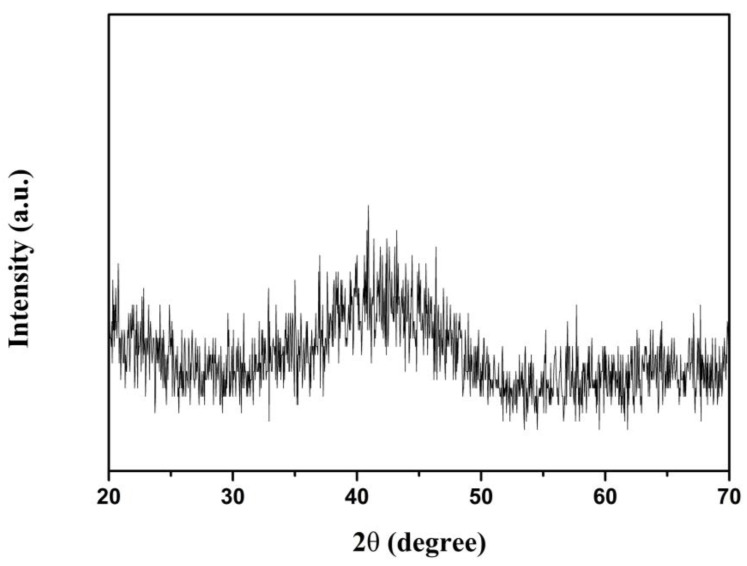
X-ray diffraction pattern of the (Nd_0.__525_Pr_0.__175_Ce_0.__3_)_9_Fe_64.__5_Co_3_Cu_0.__5_Ti_1_B_22_ as-spun ribbons.

The XRD pattern of (Nd_0.__525_Pr_0.__175_Ce_0.__3_)_9_Fe_64.__5_Co_3_Cu_0.__5_Ti_1_B_22_ as-spun ribbons is shown in Figure 1. No distinct diffraction peaks were found in the patterns, the broad peak in the interval 35–50 degrees is due to the amorphous phase.

**Figure 2:**
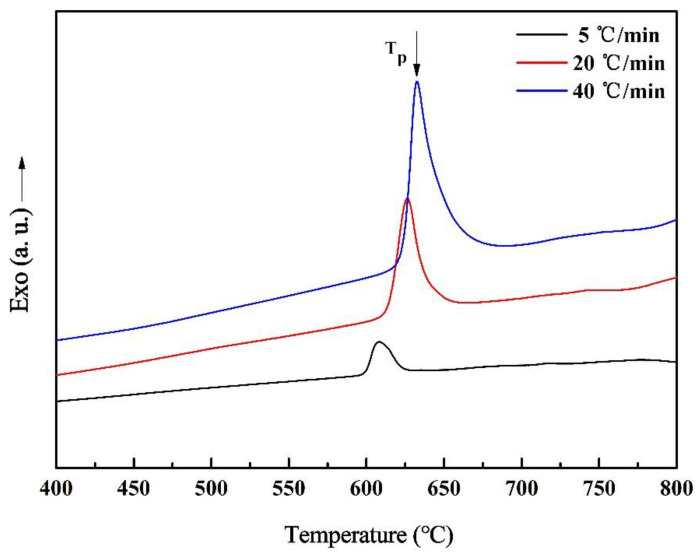
DSC curves of the (Nd_0.__525_Pr_0.__175_Ce_0.__3_)_9_Fe_64.__5_Co_3_Cu_0.__5_Ti_1_B_22_ as-spun ribbons.

**Figure 3:**
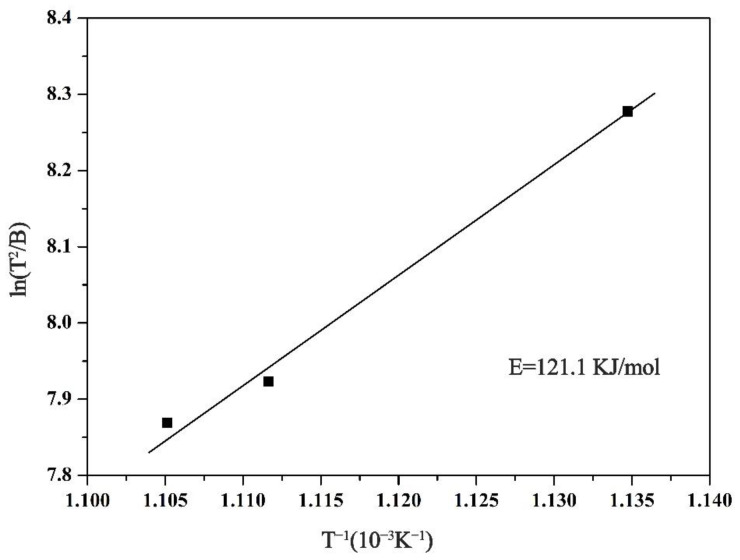
Kissinger curve of the (Nd_0.__525_Pr_0.__175_Ce_0.__3_)_9_Fe_64.__5_Co_3_Cu_0.__5_Ti_1_B_22_ as-spun ribbons.

The DSC curves of the (Nd_0.__525_Pr_0.__175_Ce_0.__3_)_9_Fe_64.__5_Co_3_Cu_0.__5_Ti_1_B_22_ as-spun ribbons at a heating rate of 5, 20, and 40 °C/min are shown in Figure 2. Only one exothermic peak is detected, which indicates the synchronized precipitation of soft and hard magnetic phases, and they were formed at the same temperature. The crystallization characteristic temperature increased with the increase of the heating rate due to the hysteresis effect of phase change and the superheat. Moreover, as the heating rate increased, the higher the phase change energy storage per unit time was, the higher the heat released during the phase change was, so the exothermic peak became larger. Activation energy during the crystallization is a crucial parameter of thermal stability for the amorphous alloy, which statistically reflects the average of the activation energy in the crystallization process. The crystallization tendency of the amorphous alloy decreases with the increase of effective activation energy. According to Kissinger equation [26]:

(1) $$In\frac{T^{2}}{B}=\frac{E}{RT}+C$$

where In is natural logarithm; *B* is the heating rate; *T* is the characteristic crystallization temperature (in this study, we select *T* = *T*_p_); E is the crystallization activation energy; *R* is the molar gas constant; and *C* is constant. The linear relationship between $\frac{T^{2}}{B}$ and $\frac{1}{T}$ is shown in Figure 3. The slope values of *E/R* represent crystallization activation energy, resulting from *E* = 121.1 KJ/mol.

### 3.2. Comparison of Conventional Annealing and Microwave Annealing

**Figure 4:**
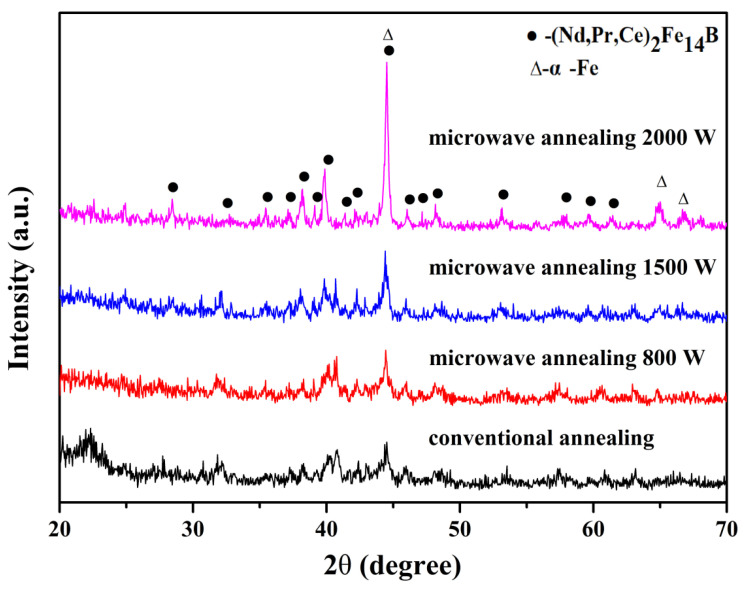
X-ray diffraction patterns of the annealed (Nd_0.__525_Pr_0.__175_Ce_0.__3_)_9_Fe_64.__5_Co_3_Cu_0.__5_Ti_1_B_22_ ribbons under different conditions.

The XRD patterns of the (Nd_0.__525_Pr_0.__175_Ce_0.__3_)_9_Fe_64.__5_Co_3_Cu_0.__5_Ti_1_B_22_ ribbons annealed at 650 °C for 10 min under conventional and microwave heating conditions, respectively, are shown in Figure 4. Apart from the diffraction peaks from (Nd, Pr, Ce)_2_Fe_14_B and α-Fe phases, a broad peak locates from 20 degrees to 25 degrees is detected, suggesting the presence of some amorphous phases after conventional annealing treatment. However, the ribbons were annealed by microwave by increasing the microwave power from 800 W to 2000 W, the intensity of the α-Fe peak became significantly stronger than the (Nd, Pr, Ce)_2_Fe_14_B peak. The grain size can be calculated from the line width (FWHM in radian) of XRD [27]. It shows that with the increase of microwave power, the grain size of the α-Fe phase increased greatly; however, the grain size of the (Nd, Pr, Ce)_2_Fe_14_B phase increased slightly, as are shown in Table 1, because microwave annealing treatment promotes the diffusion of elements, accelerates the crystallization rate, and then promotes the growth of grains, especially the α-Fe phase. The average size of grains increases from 11.9 nm in traditional annealing to 40.3 nm in microwave annealing at 2000 W. It explained that the microwave annealing treatment increased the grain size. Moreover, the broad peak completely disappears, presenting the elimination of the residual amorphous phase. The desired microstructural modifications have been achieved in the (Nd, Pr, Ce)_2_Fe_14_B/α-Fe samples after microwave annealing for the full range of power. The diffusion rates of the elements were promoted by a microwave field, the proportion of hard and soft magnetic phases were adjusted as well.

**Figure 5:**
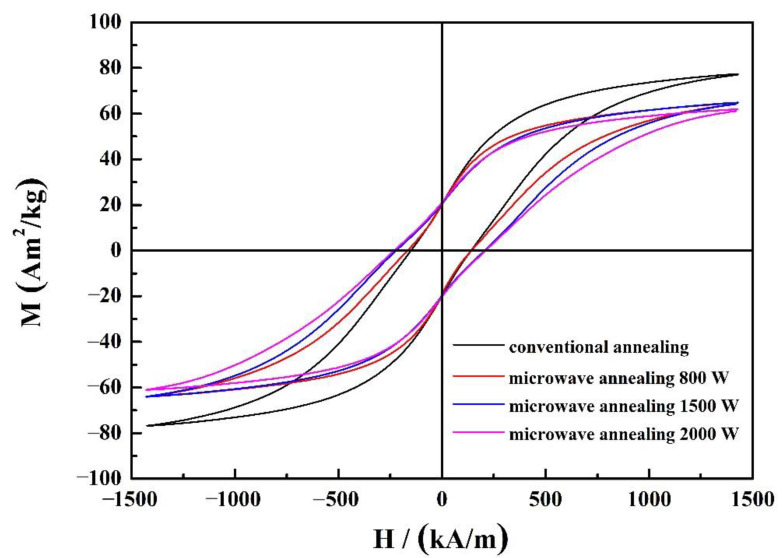
Hysteresis loop curves of the annealed (Nd_0.__525_Pr_0.__175_Ce_0.__3_)_9_Fe_64.__5_Co_3_Cu_0.__5_Ti_1_B_22_ ribbons under different conditions.

The hysteresis loop curves of the (Nd_0.__525_Pr_0.__175_Ce_0.__3_)_9_Fe_64.__5_Co_3_Cu_0.__5_Ti_1_B_22_ ribbons annealed at 650 °C for 10 min under conventional heating and microwave heating under different powers are shown in Figure 5. Their hysteresis loops display low magnetic properties. As reported, the Ce_2_Fe_14_B phase presents a lower first-order anisotropy constant than the Nd_2_Fe_14_B phase at room temperature, which induced the decrease of coercivity [28,29,30]. Even though it is expected that microwave heat treatment will increase the residual magnetization, there is no significant residual magnetic enhancement effect, and there is a weak exchange coupling effect between the soft and hard magnetic phases. The coercivity was enhanced with higher microwave power. It shows that the ribbons’ coercivity and remanence magnetic ratio under microwave annealing treatment with a power of 2000 W reaches 245 kA/m and 0.34, which are higher than that of conventional annealing (132 kA/m and 0.26), as are shown in Table 2.

**Figure 6:**
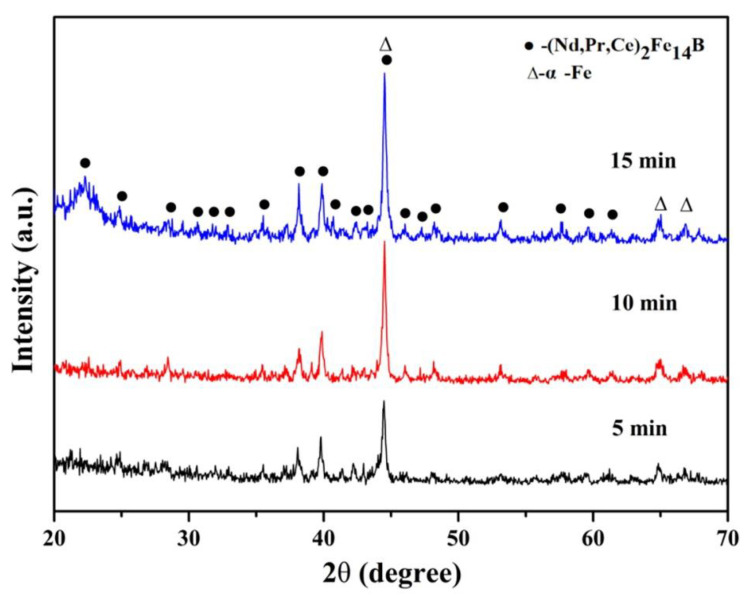
X-ray diffraction patterns of the microwave annealed (Nd_0.__525_Pr_0.__175_Ce_0.__3_)_9_Fe_64.__5_Co_3_Cu_0.__5_Ti_1_B_22_ ribbons for different time.

The X-ray diffraction patterns of the (Nd_0.__525_Pr_0.__175_Ce_0.__3_)_9_Fe_64.__5_Co_3_Cu_0.__5_Ti_1_B_22_ ribbons annealed for 5, 10, and 15 min at a microwave power of 2000 W, as are, respectively, shown in Figure 6. With the increase of crystallization time, the intensity of the pattern peaks has an increasing tendency. It shows that the grain size of the α-Fe phase increased greatly; however, the grain size of the (Nd, Pr, Ce)_2_Fe_14_B phase increased slightly, as is shown in Table 3. This is because the elements are difficult to fully diffuse in a short crystallization time, so the grains did not have enough time to grow. When annealing for 5 min, the average grain size of the α-Fe phase is only 26.9 nm. However, when the crystallization time is longer, the elements can fully diffuse, which promotes the grain growth; when annealing for 15 min, the average grain size of the α-Fe phase reached 60.2 nm. It indicated that the crystallization time affected the grain size and adjustment of the percentage of soft and hard magnetic phases.

**Figure 7:**
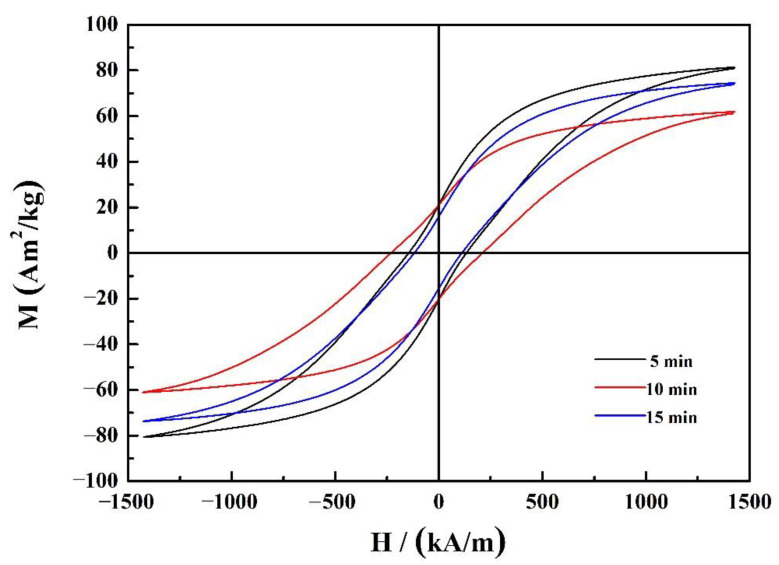
Hysteresis loop curves of the microwave annealed (Nd_0.__525_Pr_0.__175_Ce_0.__3_)_9_Fe_64.__5_Co_3_Cu_0.__5_Ti_1_B_22_ ribbons for a different time.

The hysteresis loop curves of the (Nd_0.__525_Pr_0.__175_Ce_0.__3_)_9_Fe_64.__5_Co_3_Cu_0.__5_Ti_1_B_22_ ribbons annealed for 5, 10, and 15 min at a microwave power of 2000 W, are, respectively, shown in Figure 7. It shows that annealing under the microwave power of 2000 W for 10 min could obtain a higher coercivity and residual magnetization, 245 kA/m and 21 Am^2^/kg, as are, respectively, shown in Table 4. There is no significant residual magnetic enhancement effect, and there is a weak exchange coupling effect between the soft and hard magnetic phases annealing under the microwave power of 2000 W for 10 min. The coercivity and remanent magnetic ratio could be enhanced under appropriate microwave annealing process.

## 4. Conclusions

This study investigated the crystallization and magnetic properties of (Nd, Pr, Ce)_2_Fe_14_B/α-Fe nanocomposite magnets through microwave annealing treatment. The results of the experiment indicate that compared with traditional annealing, even though microwave annealing slightly reduces the magnetic saturation strength of the magnet, it increases the coercivity and remanence ratio of (Nd, Pr, Ce)_2_Fe_14_B/α-Fe nanocomposite magnets. Coercivity and remanence ratio up to 245 kA/m and 0.38 for (Nd_0.__525_Pr_0.__175_Ce_0.__3_)_9_Fe_64.__5_Co_3_Cu_0.__5_Ti_1_B_22_ ribbons were obtained after annealed at microwave field with 2000 W for 10 min.

## Figures and Tables

**Table 1:** The average crystallites size of the main phases of the microwave annealed (Nd_0.__525_Pr_0.__175_Ce_0.__3_)_9_Fe_64.__5_Co_3_Cu_0.__5_Ti_1_B_22_ ribbons under different power applied.

Power Applied	D (nm)	
	α-Fe	(Nd, Pr, Ce)_2_Fe_14_B
conventional	11.9	9.6
800 W	17.7	12.3
1500 W	25.3	12.9
2000 W	41.3	18.6

**Table 2:** The magnetic properties of the annealed (Nd_0.__525_Pr_0.__175_Ce_0.__3_)_9_Fe_64.__5_Co_3_Cu_0.__5_Ti_1_B_22_ ribbons under different annealing conditions.

Annealing Conditions	σ_s_ (Am^2^/kg)	σ_r_ (Am^2^/kg)	H_c_ (kA/m)	σ_r_/σ_s_
conventional	78	20	132	0.26
800 W	65	20	135	0.31
1500 W	65	20	242	0.31
2000 W	61	21	245	0.34

The **σ_s_** is remanence magnetic, **σ_r_** is saturation value of magnetization, **H_c_** is coercivity, and **σ_r_/σ_s_** is remanence magnetic ratio.

**Table 3:** The average crystallites size of the main phases of the microwave annealed (Nd_0.__525_Pr_0.__175_Ce_0.__3_)_9_Fe_64.__5_Co_3_Cu_0.__5_Ti_1_B_22_ ribbons for a different time.

Annealing Time (min)	D (nm)	
	α-Fe	(Nd, Pr, Ce)_2_Fe_14_B
5	26.9	11.7
10	41.3	18.6
15	60.2	22.4

**Table 4:** The magnetic properties of the annealed (Nd_0.__525_Pr_0.__175_Ce_0.__3_)_9_Fe_64.__5_Co_3_Cu_0.__5_Ti_1_B_22_ ribbons for a different time.

Annealing Times (min)	*σ_s_* (Am^2^/kg)	*σ_r_* (Am^2^/kg)	*H_c_* (kA/m)	*σ_r_*/*σ_s_*
5	81	21	127	0.27
10	61	23	245	0.38
15	74	18	119	0.24

The ***σ_s_*** is remanence magnetic, ***σ_r_*** is saturation value of magnetization, ***H_c_*** is coercivity, and ***σ_r_*/*σ_s_*** is remanence magnetic ratio.

## Data Availability

No new data were created or analyzed in this study. Data sharing is not applicable to this article.
